# Numerical Mesoscale Analysis of Textile Reinforced Concrete

**DOI:** 10.3390/ma13183944

**Published:** 2020-09-06

**Authors:** Alexander Fuchs, Iurie Curosu, Michael Kaliske

**Affiliations:** 1 Institute for Structural Analysis, TU Dresden, 01062 Dresden, Germany; alexander.fuchs2@tu-dresden.de; 2 Institute of Construction Materials, TU Dresden, 01062 Dresden, Germany; iurie.curosu@tu-dresden.de

**Keywords:** multiscale analysis, numerical material testing, textile reinforced concrete

## Abstract

This contribution presents a framework for Numerical Material Testing (NMT) of textile reinforced concrete based on the mesomechanical analysis of a Representative Volume Element (RVE). Hence, the focus of this work is on the construction of a proper RVE representing the dominant mechanical characteristics of Textile Reinforced Concrete (TRC). For this purpose, the RVE geometry is derived from the periodic mesostructure. Furthermore, sufficient constitutive models for the individual composite constituents as well as their interfacial interactions are considered, accounting for the particular mechanical properties. The textile yarns are modeled as elastic transversal isotropic unidirectional layers. For the concrete matrix, an advanced gradient enhanced microplane model is utilized considering the complex plasticity and damage behavior at multiaxial loading conditions. The mechanical interactions of the constituents are modeled by an interface formulation considering debonding and friction as well as contact. These individual constitutive models are calibrated by corresponding experimental results. Finally, the damage mechanisms as well as the load bearing behavior of the constructed TRC-RVE are analyzed within an NMT procedure based on a first-order homogenization approach. Moreover, the effective constitutive characteristics of the composite at macroscale are derived. The numerical results are discussed and compared to experimental results.

## 1. Introduction

Textile Reinforced Concrete (TRC) is a novel and high performance cementitious composite, which consists of a textile structure made of continuous multi filament yarns embedded in a fine grained concrete matrix. Due to its high tensile strength and enhanced ductility, TRC offers innovative opportunities in design and lightweight construction, see, e.g., [1]. Moreover, its simple production makes TRC well suited for repair and strengthening of existing concrete structures [2,3,4].

The beneficial properties of this promising composite are the result of the material characteristics of the particular constituents in combination with the interactions of the constituents within the underlying mesostructure. Here, numerical investigations offer a great and unique opportunity to get detailed insight into the mesomechanical interactions in order to identify and understand the complex load bearing and damage mechanisms at arbitrary loading conditions. The identified mesostructural characteristics can be linked to the effective macroscopic composite behavior to provide a profound bridging of the different length scales. Moreover, based on a numerical model representing the mesostructure of the composite, an optimization of the effective mechanical behavior with respect to the choice of constituent materials as well as the mesostructural setup is feasible in a straightforward manner.

In the literature, several approaches to investigate and model the mechanical characteristics of TRC at different length scales can be found. In [5], a phenomenological macroscopic constitutive description based on a strain-hardening microplane damage model is presented accounting for the biaxial behavior of thin-walled TRC shells. An approach to model retrospectively strengthening of steel reinforced concrete structures with a layered textile reinforcement is described in [6,7]. There, the effective macroscopic TRC characteristics are modeled as a constitutive law within a multi reference plane approach suitable for folded plate structures. Beside such phenomenological formulations, in [8,9], a multiscale model is proposed focusing on the interaction and load transfer between concrete and a single yarn in a unidirectional composite at uniaxial loading scenarios. In these approaches, the considered composite is modeled by a structure of uniaxial bar elements, representing concrete as well as yarn filaments and which are connected to nonlinear spring elements representing the fiber matrix bond. A similar method can be found in [10], where the crack bridging behavior of a unidirectional fiber with randomly distributed mechanical properties is investigated using a uniaxial shear-lag model, as it is described, e.g., in [11,12]. Analytical descriptions, accounting for pull-out phenomena and their influence on the macroscopic behavior of unidirectional TRC, are presented in [13,14]. In [15], a hierarchical micro-meso-macro model is presented, considering and linking the relevant mechanical effects at the different scales of TRC at uniaxial tension. A similar method for the 3D multiscale analysis of TRC sandwich panels is proposed in [16].

A more general approach towards the multiaxial behavior of arbitrary complex micro and meso structures within the context of multiscale analysis is given by computational homogenization methods based on the concept of representative volume elements (RVE). Following [17] or [18], an RVE denotes a part of the micro or mesostructure which contains all relevant characteristics of the overall structure. Consequently, the RVE represents the effective macroscopic properties of the composite independent of the macroscopic location or boundary conditions.

Another important aspect is the concept of scale separation, which states that the characteristic length of the RVE must be negligibly small with respect to the characteristic length (spatial extend or fluctuations of field variables) of the macroscopic structure. Therefore, the separation of scales enables the linkage of a macroscopic material point to the corresponding RVE. Furthermore, the effective macroscopic constitutive behavior can be considered as homogeneous and can be described within the framework of continuum mechanics. From these concepts, subsequent requirements regarding the size of an RVE can be derived. For statistical investigations on the choice of an RVE, see [19]. Following [20], multiscale modeling is based on the so-called Hill-condition, which states that the stress power at a macroscopic material point is equal to the volume average of the stress power of the related RVE. Based on this condition, different boundary conditions (BC) for the analysis of the mesoscale boundary value problem can be derived. The most common types are linear displacement (LDBC), uniform traction (UTBC) and periodic boundary conditions (PBC), as it is explained in [21]. A great overview and more detailed explanations about the theoretical aspects of this type of homogenization methods can be found in [22,23,24,25]. From this theoretical basis, two different kinds of computational homogenization are derived in the literature. One is the FE${}^{2}$ approach, where the mesostructural boundary value problem is coupled to the deformation of the corresponding RVE at a certain macroscopic material point and solved in a nested finite element analysis, see, e.g., [22]. On the other hand, in [26], a decoupled homogenization method is presented, where a particular RVE is characterized in preceding numerical material tests (NMT). Subsequently, the achieved material behavior is modeled by an appropriate macroscopic constitutive law. The application of the decoupled multiscale analysis to fiber reinforced plastics is presented in [27,28]. Besides the derivation of constitutive laws, the NMT procedure provides a valuable insight into the dominant mesomechanical effects and enables sensitivity studies with respect to material as well as structural parameters. Such studies have been conducted for concrete in e.g., [29,30,31,32] and other reinforced cementitious composites in e.g., [33,34,35].

In the contribution at hand, a detailed 3D finite element model of an RVE of textile reinforced concrete is presented, taking into account the mesostructural geometry as well as the complex material behavior of the constituents and the dominant effects at the material interfaces. For this purpose, an appropriate constitutive model for concrete is utilized considering the complex mechanical characteristics at multiaxial loading conditions. Furthermore, the anisotropic linear elastic behavior of the textile yarns is modeled in a homogenized manner, based on the properties of the yarn filaments and yarn coating. The mechanical interactions at the material interfaces, such as debonding and friction, are modeled via cohesive elements with an according traction separation law (TSL). The introduced constitutive formulations are calibrated by individual experimental tests for the particular constituents. The constructed TRC-RVE is investigated within an NMT procedure in order to identify and analyze the dominant load bearing and damage mechanisms. Moreover, the macroscopic mechanical response of the RVE is evaluated in terms of effective stress–strain relations. Finally, the obtained numerical results are discussed and compared to experimental investigations.

## 2. Construction of a Representative Volume Element for Textile Reinforced Concrete

**Figure 1:**
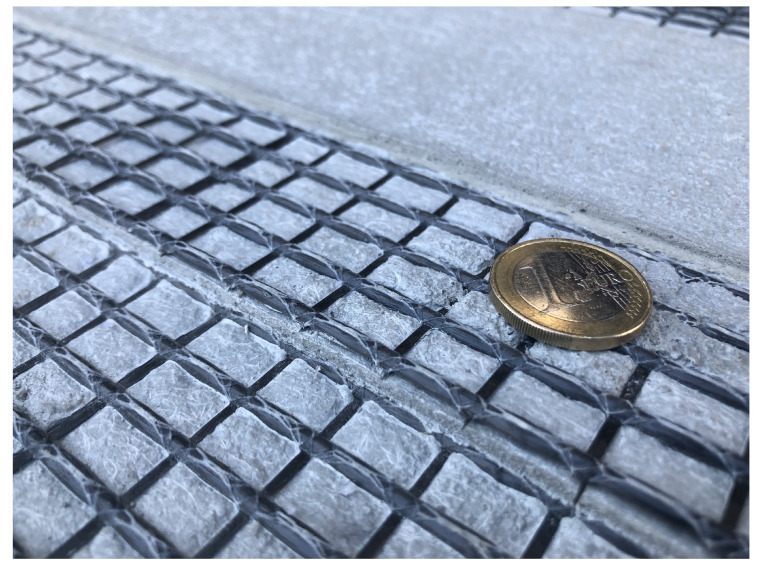
Mesostructural setup of textile reinforced concrete.

In this section, the construction of a 3D finite element model representing the mesostructure of TRC in terms of an RVE is presented. Here, the particular composite material consists of flat biaxial carbon textiles embedded in a fine grained concrete matrix according to [36]. Usually, TRC is reinforced with multiple textile layers forming plate or shell like structures in order to build lightweight or thin strengthening components, as it is shown in Figure 1. In this study, the mechanical behavior of TRC reinforced by two textile layers is investigated, according to the experimental test setup in [36,37]. It is noteworthy to mention that the shown framework for the construction of a TRC-RVE can be easily extended to an arbitrary number of reinforcement layers or adopted to other types of textile structures.

### 2.1. Geometry and Finite-Element Discretization

Due to the thin layered structure and the comparatively small aggregates of the fine grained concrete, the mesostructural setup and geometry of TRC is mainly governed by the yarn arrangement of the textile. Here, a biaxial carbon textile (TUDALIT-BZT2-V.FRAAS) with an orthogonal grid structure is considered, which consists of thick warp yarns laid on top of thin weft yarns, see Figure 1.

**Figure 2:**
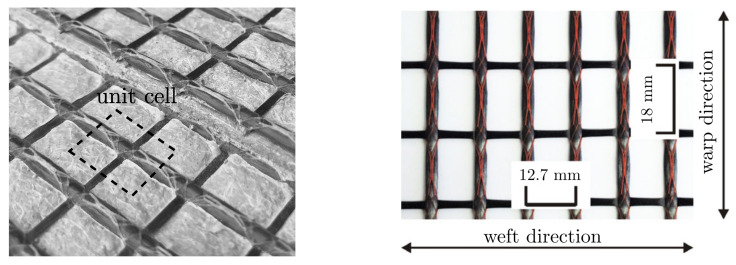
Mesostructural unit cell (**left**) and textile reinforcement with carbon filaments TUDALIT-BZT2- V.FRAAS adopted from [36] (**right**).

Furthermore, it is assumed that the concrete matrix can be modeled as a homogeneous continuum with negligible small aggregates. With this assumption, the mesostructure can be represented by a unit cell of the textile grid, as it is illustrated in Figure 2. Here, a unit cell is chosen, where one yarn crossing is embedded in the center of a small concrete block. It is worth mentioning that, for the grid structure at hand, several unit cells with an arbitrary position of the yarn-crossing could be used. The particular grid size, determining the dimensions of the unit cell, is given as 18 mm × 12.7 mm, as shown in Figure 2.

**Figure 3:**
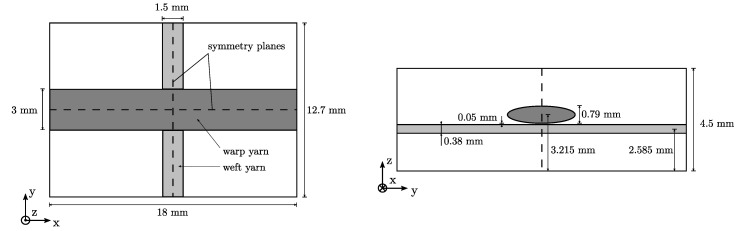
Geometry of a single layer TRC-RVE: top view (**left**), side view (**right**).

The cylindrical yarns are modeled by an elliptical cross-section and a cross-section area of $A_{\mathrm{warp}}=1.8$ mm${}^{2}$ and $A_{\mathrm{weft}}=0.45$ mm${}^{2}$ according to [36]. Consequently, the volume fractions with respect to the volume of the RVE yield $\psi_{\mathrm{warp}}=0.0315$ and $\psi_{\mathrm{weft}}=0.0055$. In order to avoid poorly shaped elements in the regions of the yarn-crossings, a small spacing of 0.05 mm is introduced. The final geometry of the TRC-RVE is illustrated in Figure 3.

**Figure 4:**
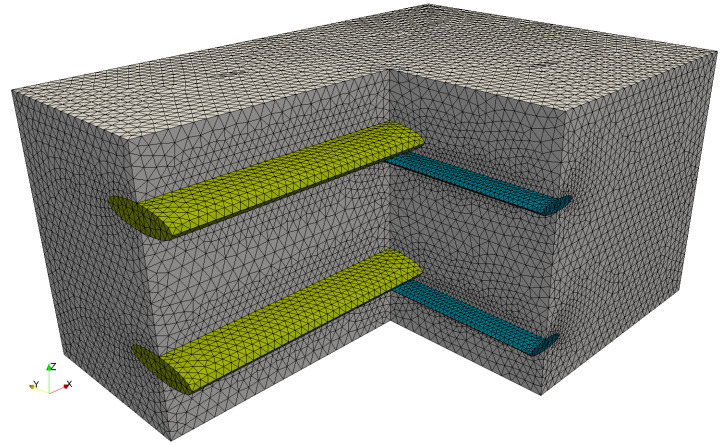
Finite element mesh of two layered TRC-RVE with 4 × 143,492 linear tetrahedral elements, 4 × 3700 cohesive zone elements and 4 × 28,126 nodes.

Based on the derived geometry, the RVE is meshed by linear tetrahedral finite elements. Since the RVE represents a periodic structure, periodic boundary conditions are most appropriate to load and study the RVE. Basically, PBC link or enforce the same value for the degrees of freedom of matching nodes on opposite faces of the RVE in order to consider the behavior of adjacent unit cells. For this purpose, the nodes of opposite boundaries must be coincident and form a so-called periodic boundary. It is noteworthy to mention that, in the literature, techniques for imposing PBC on arbitrary meshes exist, e.g., [38]. These methods are based on polynomial approximations and require higher computational effort. Thus, a periodic boundary mesh is preferable. For the considered TRC-RVE, the mesostructural periodicity exists only in-plane of the layered structure. Therefore, PBC can only be imposed on the boundaries perpendicular to the grid. To generate a proper boundary mesh, a quarter of the RVE is firstly meshed and then mirrored two times. Composites with multiple textile layers can be modeled by stacking RVE of one layer on each other. Please note that in such cases the node positions of touching boundaries must be modified accordingly. Here, a two layered TRC-RVE with a subsequent thickness of 9 mm, according to [36,37], is considered. Finally, cohesive zone elements are inserted at the material interfaces. The created FE mesh used in this study is shown in Figure 4.

### 2.2. Constitutive Modeling of Composite Constituents

The generated RVE consists essentially of three different materials (concrete, warp yarn, and weft yarn) connected by cohesive zone elements. In order to capture the relevant mesomechanical effects, appropriate constitutive models are required, describing the relevant material characteristics of the particular constituent as well as their interactions. In this section, the key features of the utilized constitutive models are pointed out and their mathematical formulation is briefly explained. Please note that all of the following constitutive models are formulated within the theory of small strain.

#### 2.2.1. Textile Yarns

The yarns of a textile reinforcement layer consist of a bundle of filaments impregnated by a polymeric coating. Hence, the textile yarns are a composite material itself. In this study, the yarns are considered as a unidirectional (UD) layer of fiber reinforced plastic (FRP) with homogeneous effective properties at the mesoscale. One approach for the identification of these mesoscopic effective characteristics is a detailed microscale analysis of the yarn, as it is shown e.g., in [39]. In this work, a phenomenological approach for the effective yarn behavior is used, according to [40]. However, it is known from experiments that the carbon yarns show very brittle behavior with approximately linear elastic stress–strain relation up to 1700 MPa in fiber direction, see [41]. Therefore, the yarns are modeled as transversal isotropic linear elastic UD layer with the longitudinal direction as the preferential direction. With this assumption, the effective elastic properties can be determined based on an empirically modified rule of mixture, as it is described in [40]. Consequently, the stiffness matrix of a yarn in Voigt notation can be written as

(1) $$C={\left[\begin{array}{cccccc} \frac{1}{E_{⊥}} & -\frac{\nu_{⊥⊥}}{E_{⊥}} & -\frac{\nu_{⊥\|}}{E_{\|}} & 0 & 0 & 0\\ -\frac{\nu_{⊥⊥}}{E_{⊥}} & \frac{1}{E_{⊥}} & \frac{\nu_{⊥\|}}{E_{\|}} & 0 & 0 & 0\\ -\frac{\nu_{\|⊥}}{E_{⊥}} & -\frac{\nu_{\|⊥}}{E_{⊥}} & \frac{1}{E_{\|}} & 0 & 0 & 0\\ 0 & 0 & 0 & \frac{1}{G_{⊥⊥}} & 0 & 0\\ 0 & 0 & 0 & 0 & \frac{1}{G_{⊥\|}} & 0\\ 0 & 0 & 0 & 0 & 0 & \frac{1}{G_{⊥\|}} \end{array}\right]}^{-1},$$

where *E* denotes the Young’s modulus, ν the Poisson’s ratio, and *G* the shear modulus. The indices || and ⊥ refer to the different longitudinal and transversal direction of the yarn or filaments, respectively. Please note that, in Equation (1), the preferential or longitudinal direction is oriented along the third global axis. The orientation of the preferential direction can be easily changed by a corresponding transformation operation. Following [40], the effective elastic parameters in Equation (1) can be computed based on the elastic properties of the transversal isotropic filaments (indicated by *f*), the isotropic polymeric matrix (indicated by *m*) and the fiber volume fraction φ as follows:

(2) $$\begin{array}{cc} & E_{\|}=E_{f\|}\cdot \varphi +E_{m}\cdot \left(1-\varphi \right), \end{array}$$

(3) $$\begin{array}{cc} & E_{⊥}=\frac{E_{m}}{1-\nu_{m}^{2}}\cdot \frac{1+0.85\cdot \varphi^{2}}{{\left(1-\varphi \right)}^{1.25}+\varphi \cdot \frac{E_{m}}{\left(1-\nu_{m}^{2}\right)\cdot E_{f⊥}}}, \end{array}$$

(4) $$\begin{array}{cc} & G_{⊥\|}=G_{m}\frac{1+0.4\cdot \varphi^{0.5}}{{\left(1-\varphi \right)}^{1.45}+\frac{G_{m}}{G_{f}}\cdot \varphi }, \end{array}$$

(5) $$\begin{array}{cc} & \nu_{⊥\|}=\varphi \cdot \nu_{f⊥\|}+\nu_{m}\cdot \left(1-\varphi \right), \end{array}$$

(6) $$\begin{array}{cc} & \nu_{\|⊥}=\nu_{⊥\|}\cdot \frac{E_{⊥}}{E_{\|}}, \end{array}$$

(7) $$\begin{array}{cc} & \nu_{⊥⊥}=\varphi \cdot \nu_{f⊥⊥}+\left(1-\varphi \right)\cdot \nu_{m}\frac{1+\nu_{m}-\nu_{f}\cdot E_{m}/E_{f\|}}{1-\nu_{m}^{2}+\nu_{m}\cdot \nu_{f}\cdot E_{m}/E_{f\|}}, \end{array}$$

(8) $$\begin{array}{cc} & G_{⊥⊥}=\frac{E_{⊥}}{2(1+\nu_{⊥⊥})}. \end{array}$$

#### 2.2.2. Concrete

For the constitutive formulation of the fine grained concrete matrix, a gradient enhanced viscoplasticity damage microplane model for concrete is utilized, adopted from [42]. As mentioned before, the concrete matrix is considered as homogeneous, since the aggregates are assumed to be negligibly small. The adopted concrete model accounts for the complex material behavior at multiaxial loading states with special focus on dominant plasticity and damage effects as well as induced anisotropy. In the following, the key features and their mathematical formulation are explained. Please note that here the viscoplasticity formulation presented in [42] is reduced to a plasticity formulation without viscous or rate effects, since the study at hand accounts for the quasi static behavior of TRC.

In the literature, several microplane models with different stress and strain projections as well as constitutive laws are proposed. Here, the model is formulated in a thermodynamic consistent manner based on the kinematic constraint, i.e., the microplane strains are assumed to be equal to the projection of the mesoscopic strain tensor to the microplanes. In addition, a volumetric-deviatoric (V-D) split of the microplane strain and stress quantities is utilized, with the related projection tensors V and Dev, respectively. Hence, the relation between the mesoscopic strain tensor ϵ and the microplane strains is given as

(9) $$\epsilon_{V}=V:\epsilon \;,\;\epsilon_{D}=\mathrm{Dev}:\epsilon .$$

By decomposing the microplane strains in an elastic and a plastic part and taking damage into account by a scalar damage parameter $d^{mic}$ applied to both the volumetric and deviatoric stress components, the mesoscopic stress–strain relation reads

(10) $$\sigma =\frac{3}{4\pi }\int_{\Omega }\left(1-d^{mic}\right)\left[K^{mic}V\left(\epsilon_{V}-\epsilon_{V}^{pl}\right)+2G^{mic}{\mathrm{Dev}}^{T}\cdot \left(\epsilon_{D}-\epsilon_{D}^{pl}\right)\right]\;d\Omega ,$$

where $K^{mic}$ and $G^{mic}$ are the microplane bulk and shear moduli, respectively. The integration over the surface of the microplane unit sphere, denoted as Ω, is done by a numerical integration scheme using 21 independent microplanes, according to [43]. The flow rules, describing the evolution of the microplane plastic strains, are given as

(11) $${\dot{\epsilon }}_{V}^{pl}=\dot{\lambda }m_{V}\;,\;{\dot{\epsilon }}_{D}^{pl}=\dot{\lambda }m_{D},$$

where λ is a plastic multiplier. The respective flow directions $m_{V}$ and $m_{D}$ are derived from the microplane yield function $f^{mic}$ as follows:

(12) $$m_{V}=\frac{\partial f^{mic}}{\partial \sigma_{V}}\;,\;m_{D}=\frac{\partial f^{mic}}{\partial \sigma_{D}}.$$

With these definitions, the microplane stresses read

(13) $$\begin{array}{cc} \sigma_{V} & =K^{mic}\left(\epsilon_{V}-\epsilon_{V}^{pl}\right), \end{array}$$

(14) $$\begin{array}{cc} \sigma_{D} & =2G^{mic}\left(\epsilon_{D}-\epsilon_{D}^{pl}\right). \end{array}$$

**Figure 5:**
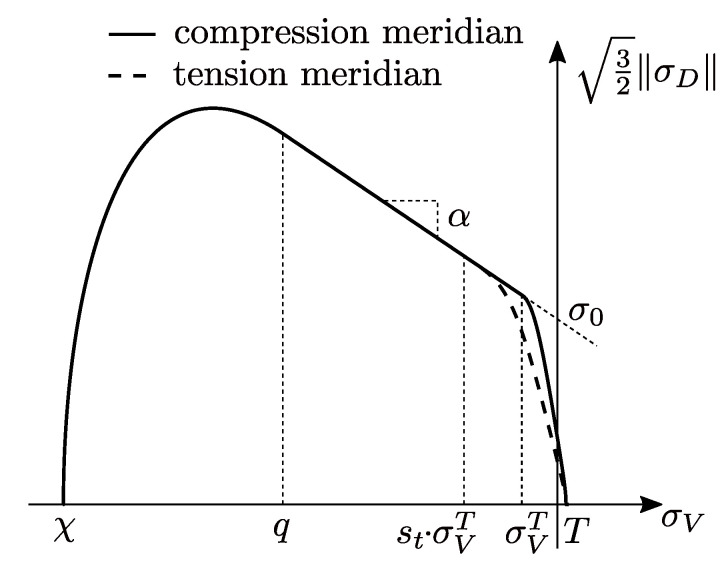
Initial smooth microplane cap yield function.

The microplane bulk modulus $K^{mic}$ and shear modulus $G^{mic}$ can be derived from their mesoscopic counterparts as $K^{mic}=3K$ and $G^{mic}=G$. To capture the complex triaxial behavior of concrete, a smooth microplane cap yield function is used. This yield function is expressed in terms of the microplane stresses and consists of a Drucker–Prager function enhanced by a tension and a compression cap. Furthermore, to distinguish and account for the different material behaviors at multiaxial loading states, the third stress invariant $J_{3}$ is considered. This invariant characterizes the multiaxial loading state and enables a formulation, which distinguishes between triaxial tension and compression. The utilized yield function is shown in Figure 5 and is formulated as

(15) $$f^{mic}=\frac{3}{2}\sigma_{D}\cdot \sigma_{D}-f_{1}^{2}\;f_{t}\;f_{c}+f_{P},$$

where $f_{t}$ and $f_{c}$ are tension and compression caps, respectively. The microplane Drucker–Prager function $f_{1}$ is given as

(16) $$f_{1}=\sigma_{0}-\alpha \sigma_{V}+f_{h}\left(\kappa \right),$$

where $\sigma_{0}$ is the initial yield stress and α is the friction coefficient. Furthermore, a linear isotropic hardening function $f_{h}$ is used, which is driven by the corresponding internal variable κ and reads

(17) $$f_{h}\left(\kappa \right)=D_{h}\kappa ,$$

where $D_{h}$ is a hardening parameter. The evolution of κ is simply defined as

(18) $$\dot{\kappa }=\dot{\lambda }.$$

The compression cap is formulated as

(19) $$\begin{array}{cc} f_{c} & =1-H_{c}\left(q-\sigma_{V}\right)\frac{{\left(\sigma_{V}-q\right)}^{2}}{\chi^{2}}, \end{array}$$

(20) $$\begin{array}{cc} \chi & =R\;f_{1}\left(q\right), \end{array}$$

where *R* is the volumetric-deviatroric axis ratio. The internal variable *q* describes the intersection point between the Drucker–Prager function and the compression cap. This internal variable is the driving force for the introduced cap hardening mechanisms, which describes the widening or movement of the compression cap, while the other parts of the yield function remain the same. This hardening mechanism represents the densification of concrete at high volumetric pressure, according to the Hugoniot curve of the material. Basically, the Hugoniot curve describes the nonlinear relation between the volumetric strain and hydrostatic pressure of a porous material, such as concrete, due to pore collapse and the successive compaction. Here, this nonlinear relation is approximated in terms of the volumetric plastic strains and the internal variable *q* and reads

(21) $$\epsilon_{V}^{pl}=h_{q}\left(q\right)=W\left(e^{D_{c}\left(\chi \left(q\right)-\chi_{0}\right)}-1\right),$$

where *W* is the maximum volumetric plastic strain at hydrostatic compression and $D_{c}$ is a material constant. Furthermore, χ(q) describes the current volumetric abscissa of the compression cap (see Figure 5) depending on *q*. Please note that Equation (21) also depends on the volumetric stress $\sigma_{V}$ as well as κ, due to the coupling in Equations (16) and (20). From Figure 5, it is obvious that an initial value of q=0 MPa is not meaningful. Hence, an initial value of $q=\sigma_{V}^{C}$ with the corresponding $\chi_{0}=\chi \left(\sigma_{V}^{C}\right)$ is defined, where $\sigma_{V}^{C}$ is a material constant. Finally, the evolution law of *q* is formulated in terms of the approximated Hugoniot curve in Equation (21), which yields

(22) $$\dot{q}=\dot{\lambda }\;H_{c}\frac{\partial f^{mic}}{\partial \sigma_{V}}\frac{\partial h_{q}}{\partial q}=\dot{\lambda }m_{q}.$$

The tension cap of the yield function is defined as

(23) $$\begin{array}{cc} f_{t} & =1-H_{t}\left(\sigma_{V}-s_{t}\;\sigma_{V}^{T}\right)\cdot {\left(\frac{\sigma_{V}-s_{t}\;\sigma_{V}^{T}}{T-s_{t}\;\sigma_{V}^{T}}\right)}^{2+A_{s}\frac{\sigma_{V}^{e}-\sigma_{V}^{T}}{T-\sigma_{V}^{T}}}, \end{array}$$

(24) $$\begin{array}{cc} T & =T_{0}+R_{t}f_{h}\left(\kappa \right), \end{array}$$

where $\sigma_{V}^{T}$, $R_{t}$, $T_{0}$, and $A_{s}$ are material parameters. The Heavyside functions $H_{c}$ and $H_{t}$ in the formulation of the cap functions are given as

(25) $$H\left(x\right)=\frac{1}{2}\left(1+\mathrm{sgn}\left(x\right)\right),$$

activating the particular cap when the stress state is within their domain. However, while $\sigma_{V}^{T}$ describes the initial volumetric stress at the intersection point between the tension cap and the Drucker–Prager function, $T_{0}$ represents the initial intersection of the tension cap with the volumetric axis, see Figure 5. Due to isotropic hardening, the current intersection point *T* (hydrostatic tension) can increase, where the influence of the hardening on *T* is controlled by the parameter $R_{t}$. Furthermore, the shape parameter $A_{s}$ controls the steepness of the tension cap in order to achieve a more accurate yield envelope at biaxial loading regarding the tension compression asymmetry of concrete. In order to distinguish between triaxial tension and compression loading states, the third stress invariant $J_{3}$ as well as the Lode angle θ are taken into account and read

(26) $$\theta =\frac{1}{3}\cos^{-1}\left(\frac{3\sqrt{3}\;J_{3}}{2\;J_{2}^{3/2}}\right).$$

In the formulation at hand, $J_{3}$ and θ are computed from the mesoscopic stress tensor of the previous time step, since no microplane representation of $J_{3}$ or θ is defined. Based on the Lode angle, a scaling function $s_{t}$ is introduced, which shifts the initial intersection point $\sigma_{V}^{T}$ depending on the triaxial loading state and is defined as

(27) $$\begin{array}{cc} s_{t} & =-\frac{T_{0}}{\sigma_{V}^{T}}{\left(\frac{1}{r{\left(\theta \right)}^{2}\;r_{0}}-1\right)}^{1/s_{t0}}, \end{array}$$

(28) $$\begin{array}{cc} s_{t0} & =2+A_{s}\frac{-\sigma_{V}^{T}}{T_{0}-\sigma_{V}^{T}}, \end{array}$$

(29) $$\begin{array}{cc} r_{0} & =1-{\left(\frac{-\sigma_{V}^{T}}{T_{0}-\sigma_{V}^{T}}\right)}^{s_{t0}}. \end{array}$$

With the shift of the intersection point, the steepness of the tension cap changes, which leads to a changed (decrease, see Figure 5) yield strength at a fixed volumetric stress state. Therefore, this formulation enables the consideration of the influence of the triaxialty of the loading state by the Lode angle on the yield strength. In other words, the cross-section of the effective yield function at a particular $\sigma_{V}$ can be scaled from a circle to any other shape expressed by r(θ). Here, $s_{t}$ is constructed in such a way that the cross-section in the π-plane ($\sigma_{V}=0$ MPa) is forced to be exactly the by r(θ) prescribed shape, while the influence of the scaling function decreases with increasing volumetric stresses and vanishes in the intersection point. Consequently, the Drucker–Prager part and the compression cap have always a circular cross-section. Here, the Willam-Warnke approach, see [44], for r(θ) is adopted and reads

(30) $$r\left(\theta \right)=\frac{2\left(1-e^{2}\right)\cos\left(\theta \right)+\left(2e-1\right)\sqrt{4\left(1-e^{2}\right)\cos^{2}\left(\theta \right)+5e^{2}-4e}}{4\left(1-e^{2}\right)\cos^{2}\left(\theta \right)+{\left(2e-1\right)}^{2}},$$

where e∈[0.5,1] is an eccentricity parameter.

However, the presented mathematical formulation of the tension cap might lead to non-physical results because it creates a second domain of valid stress states as a side effect, which allows stress states with higher volumetric stresses than *T*. In order to suppress this non-physical domain, a penalty function is added to the yield function and is defined as

(31) $$f_{P}=-P\;H_{P}\left(\sigma_{V}-T\right){\left(\sigma_{V}-T\right)}^{2},$$

where *P* is a penalty parameter. Note that the penalty function does not affect the other parts of the yield function, since it is only activated for states where $\sigma_{V}>T$, controlled by the corresponding Heavyside function $H_{P}$.

Due to the multiplicative coupling of the Drucker–Prager function and the proposed cap functions, a smooth $C^{1}$-continuous microplane yield surface is achieved, which offers several numerical advantages. With the introduced governing equations of the plasticity formulation, the states of the internal variables and the undergraded microplane stress states are computed by a return mapping algorithm under the consideration of the corresponding Kuhn–Tucker conditions

(32) $$\dot{\lambda }\geq 0,\;f^{mic}\leq 0,\;\dot{\lambda }\;f^{mic}=0\;.$$

For the modeling of damage initiation and evolution in concrete at monotonic and cyclic loading, it is necessary to consider the different damage characteristics in tension and compression as well as the transition between these states. Here, a damage model introduced in [43] is used, which decomposes the effective damage $d^{mic}$ into a compression $d_{c}^{mic}$ and a tension part $d_{t}^{mic}$ as follows:

(33) $$1-d^{mic}=\left(1-d_{c}^{mic}\right)\left(1-r_{w}d_{t}^{mic}\right),$$

where both parts are formulated by an exponential evolution law, reading

(34) $$\begin{array}{cc} d_{t}^{mic} & =1-\exp(-\beta_{t}\gamma_{t}^{mic}), \end{array}$$

(35) $$\begin{array}{cc} d_{c}^{mic} & =1-\exp(-\beta_{c}\gamma_{c}^{mic}). \end{array}$$

The material parameters $\beta_{t}$ and $\beta_{c}$ control the development of the related damage part. Furthermore, the split weight factor $r_{w}$ describes the transition between tension and compression states and is defined as

(36) $$r_{w}=\frac{\sum_{I=1}^{3}\langle \epsilon^{I}\rangle }{\sum_{I=1}^{3}\left|\epsilon^{I}\right|},$$

where $\langle \epsilon^{I}\rangle$ is the positive part of the *I*-th mesoscopic principal strain.

The implicit gradient enhancement, used in this approach, can be seen as a regularization method in order to overcome localization issues by a spatial averaging of a local variable $\eta_{m}$. This averaged value ${\bar{\eta }}_{m}$ is considered as an extra nonlocal degree of freedom and can be described by a partial differential equation in addition to the balance of linear momentum. Hence, the strong coupled field problem reads

(37) $$\begin{array}{cc} & \nabla \cdot \sigma +f=0, \end{array}$$

(38) $$\begin{array}{cc} & {\bar{\eta }}_{m}-c\nabla^{2}{\bar{\eta }}_{m}=\eta_{m}, \end{array}$$

where σ is the Cauchy stress tensor and f is the body force vector. The nonlocal interaction is controlled by the gradient parameter *c*. Moreover, homogeneous Neumann boundary conditions are imposed to the nonlocal field. As it can be seen from Equation (38), the evolution of the nonlocal variable ${\bar{\eta }}_{m}$ is driven by its local counter counterpart $\eta_{m}$ as a source term. In this formulation, the local variables are the homogenized effective strains

(39) $$\eta_{m}=\left[\begin{array}{c} \eta_{mt}\\ \eta_{mc} \end{array}\right]=\left[\begin{array}{c} \frac{1}{4\pi }\int_{\Omega }\eta_{t}^{mic}\;d\Omega \\ \frac{1}{4\pi }\int_{\Omega }\eta_{c}^{mic}\;d\Omega \end{array}\right],$$

which are computed from the local microplane effective strains $\eta^{mic}$. The evolution of the local microplane effective strains is based on the rate of the volumetric plastic strains and defined as

$${\dot{\eta }}_{t}^{mic}=\{\begin{array}{lll} r_{w}{\dot{\epsilon }}_{V}^{pl} & \mathrm{if}\;{\dot{\epsilon }}_{V}^{pl}>0 & \;\;\;\;\;\;\;\;\;\;\;\;\;(40)\\ 0 & \mathrm{if}\;{\dot{\epsilon }}_{V}^{pl}\leq 0 & \;\;\;\;\;\;\;\;\;\;\;\;\;(41) \end{array}$$

$${\dot{\eta }}_{c}^{mic}=\{\begin{array}{lll} (1-r_{w}){\dot{\epsilon }}_{V}^{pl} & \mathrm{if}\;{\dot{\epsilon }}_{V}^{pl}>0 & \;\;\;\;\;\;\;\;\;\;\;(42)\\ 0 & \mathrm{if}\;{\dot{\epsilon }}_{V}^{pl}\leq 0 & \;\;\;\;\;\;\;\;\;\;\;(43) \end{array}$$

As a consequence of the described gradient enhancement, two additional nonlocal degrees of freedom are introduced. However, in order to achieve a full regularization of plastic damage models, an over-nonlocal formulation is utilized, where the damage driving variable in Equations (34) and (35) is based on the over-nonlocal variable ${\hat{\eta }}^{mic}$ and reads

$$\gamma_{t}^{mic}=\{\begin{array}{lll} {\hat{\eta }}_{t}^{mic}-\gamma_{t0} & \mathrm{if}\;{\hat{\eta }}_{t}^{mic}>\gamma_{t0} & \;\;\;\;\;\;\;\;\;\;\;(44)\\ 0 & \mathrm{if}\;{\hat{\eta }}_{t}^{mic}\leq \gamma_{t0} & \;\;\;\;\;\;\;\;\;\;\;(45) \end{array}$$

$$\gamma_{c}^{mic}=\{\begin{array}{lll} {\hat{\eta }}_{c}^{mic}-\gamma_{c0} & \mathrm{if}\;{\hat{\eta }}_{c}^{mic}>\gamma_{c0} & \;\;\;\;\;\;\;\;\;\;\;(46)\\ 0 & \mathrm{if}\;{\hat{\eta }}_{c}^{mic}\leq \gamma_{c0}, & \;\;\;\;\;\;\;\;\;\;\;(47) \end{array}$$

where $\gamma_{t0}$ and $\gamma_{c0}$ are damage the threshold for tension and compression, respectively. The corresponding over-nonlocal variables are defined as a linear combination of the respective local and nonlocal variable as follows:

(48) $$\begin{array}{cc} {\hat{\eta }}_{t}^{mic} & =m{\bar{\eta }}_{mt}+\left(1-m\right)\eta_{t}^{mic}, \end{array}$$

(49) $$\begin{array}{cc} {\hat{\eta }}_{c}^{mic} & =m{\bar{\eta }}_{mc}+\left(1-m\right)\eta_{c}^{mic}. \end{array}$$

In order to achieve regularization, the material constant *m* should be larger than 1.

Finally, the presented constitutive model for concrete is implemented within a sufficient finite element formulation, following the algorithmic treatment described in [43].

#### 2.2.3. Yarn–Concrete Interface

The mechanical interactions at the material interfaces of a composite are essential for the local and, hence, the effective load transfer as well as failure mechanisms. For this reason, a sufficient description of the interfacial properties for the particular material combination is necessary. In contrast to the models proposed in [8,13,14], which describe the interfacial behavior by bond-slip laws in an integral manner and restricted to uniform pull-out scenarios, a local formulation by cohesive zone elements with an appropriate traction-separation law (TSL) is utilized in this work. The formulation of the cohesive zone elements within the framework of the finite element method is based on [45] and adopted to small strain mechanical problems. The used TSL follows the work of [46,47,48,49] and takes into account the local three-dimensional damage (debonding), friction and contact effects at the yarn concrete interface. Moreover, it considers the influence of the initial contact pressure on the frictional behavior, caused by shrinkage of concrete during the drying process, as well as crack closing effects. The consideration of these local mechanisms is of great importance in the case of non-uniform and partial debonding in combination with frictional effects of the textile yarns, as it occurs e.g., at shear loading. It is noteworthy to mention that the yarns are assumed to have a smooth surface without geometrical interlocking effects. In the following, the mathematical formulation of the cohesive zone elements as well as the TSL are briefly outlined.

For this purpose, a continuum body $B\subset R^{3}$ with a boundary $\partial B\subset R^{2}$ and an interface $\partial \Omega \subset R^{2}$ of two different materials is considered. At the interface of these two materials, a three-dimensional cohesive element with zero thickness is considered. This element consists of two opposite 4 node facets with areas $\Omega^{+}$ and $\Omega^{-}$, attached to the corresponding surface of the interacting materials. The opening of the interface is described by a separation vector s, which measures the relative displacements of two points of the interacting faces. The separation within the cohesive element can be computed by the nodal displacements $d^{e}$ as

(50) $$s=\left[N_{\Omega^{+}}-N_{\Omega^{-}}\right]d^{e},$$

where $N_{\Omega^{+}}$ and $N_{\Omega^{-}}$ are the shape functions for each facet of the cohesive element. With these definitions, the kinetic equilibrium of the regarded continuum reads

(51) $$\int_{B}\nabla N^{T}\sigma dV-\int_{\partial B}N^{T}t\;dA-\int_{\partial \Omega^{+}}N_{\Omega^{+}}^{T}t^{+}d\Omega^{+}-\int_{\partial \Omega^{-}}N_{\Omega^{-}}^{T}t^{-}d\Omega^{-}=0\;.$$

Due to zero thickness of the element, the nodes of the opposite facets are initially coincident. Therefore, the surface areas of both sides are equal $d\Omega =d\Omega^{+}=d\Omega^{-}$. Furthermore, due to the equilibrium conditions, the tractions at the opposite faces are equal $t_{IF}=-t^{+}=t^{-}$. As a consequence, the resulting interfacial force is defined as

(52) $$f_{IF}=\int_{\partial \Omega^{+}}N_{\Omega^{+}}^{T}t^{+}d\Omega^{+}+\int_{\partial \Omega^{-}}N_{\Omega^{-}}^{T}t^{-}d\Omega^{-}=\int_{\partial \Omega }\left[N_{\Omega^{+}}^{T}+N_{\Omega^{-}}^{T}\right]t_{IF}\;d\Omega \;.$$

**Figure 6:**
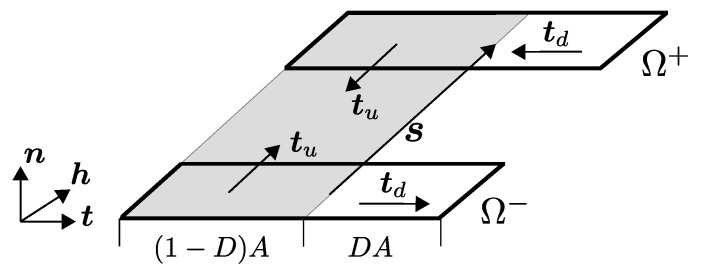
Schematic illustration of representative interface area (RIA).

The constitutive behavior of the cohesive zone is described by an appropriate TSL, which denotes the mathematical relation of the tractions as a function of the separation vector $t_{IF}=t_{IF}\left(s\right)$. Following [47], this relation is based on a representative interface area (RIA), as illustrated in Figure 6.

It is assumed that the area *A* of the RIA can be partitioned in an undamaged part $A_{u}$ and a damaged part $A_{d}$ according to the damage parameter $D=A_{d}/A$. Thus, the following relationship holds:

(53) $$A=A_{u}+A_{d}\;\mathrm{with}\;A_{d}=DA\;,\;A_{u}=\left(1D\right)A.$$

Furthermore, the separation is assumed to be constant over the whole RIA $s=s_{u}=s_{d}$. To distinguish between normal and tangential relative displacements of the RIA faces, the separation vector, which is defined in the global coordinate system, is projected into the local RIA coordinate system by

(54) $$s=\left[\begin{array}{c} s_{n}\\ s_{t}\\ s_{h} \end{array}\right]=\left[\begin{array}{c} s\cdot n\\ s\cdot t\\ s\cdot h \end{array}\right]\;.$$

The projection vectors are computed from the orthogonal reference unit vectors {N, $T_{x}$, $T_{y}$} and the local normal unit vector of the RIA as

(55) $$h=\frac{N\times n}{\left||N\times n|\right|}\;,\;t=n\times h\;.$$

However, the TSL at hand is formulated within a thermodynamically consistent setting, where the free energy per unit area is defined as a function of the damage variable *D* and reads

(56) $$\psi_{RIA}=\left(1D\right)\psi_{u}+D\psi_{d}.$$

$\psi_{u}$ and $\psi_{d}$ refer to the free energy of the damaged and undamaged part of the RIA, respectively. Consequently, the traction within the RIA can be derived as

(57) $$t_{IF}=\left(1D\right)\cdot t_{u}+D\cdot t_{d}=\left(1D\right)\left[\begin{array}{c} \sigma_{un}\\ \tau_{ut}\\ \tau_{uh} \end{array}\right]+D\left[\begin{array}{c} \sigma_{dn}\\ \tau_{dt}\\ \tau_{dh} \end{array}\right]\;.$$

While the undamaged part is considered as purely elastic, the separation in the damaged area is split into an elastic and an inelastic contribution as $s=s_{de}+s_{di}$. Hence, the elastic tractions in the undamaged part are described as

(58) $$t_{u}=Ks,$$

where $K=\mathrm{diag}(K_{n},K_{t},K_{h})$ contains the corresponding interfacial stiffnesses in the normal direction $K_{n}$ and in the tangential directions $K_{t}=K_{h}$. The tractions of the damaged part are given as

(59) $$t_{d}=Ks_{de}=K\left(s-s_{di}\right).$$

In order to prevent penetration of the opposite faces of the cohesive zone, where $s_{n}\leq 0$, the normal stiffness of the whole RIA is increased by a penalty factor $p_{c}$ and reads

$$K_{n}=\{\begin{array}{ccc} \left(60\right) & K_{n}, & \mathrm{if}\;s_{n}>0\\ \left(61\right) & p_{c}\times K_{n}, & \mathrm{if}\;s_{n}\leq 0 \end{array}$$

In this study, $p_{c}$ is set to $10^{4}$. Furthermore, the normal traction component of the damaged RIA part is considered to be active in the case of penetration and is described as

(62) $$\sigma_{dn}=K_{n}\;{\langle s_{n}\rangle }_{-}\;,$$

with the MacCauley brackets ${\langle \cdot \rangle }_{\pm }=\frac{\left(\cdot )\pm |(\cdot )\right|}{2}$. The tangential traction components $\tau_{dt}$ and $\tau_{dh}$ of $A_{d}$ are calculated from the frictional response of the RIA and are dependent on the evolution of the inelastic parts of the separation $s_{di}$. Here, the frictional behavior is modeled as a standard plasticity problem with a three-dimensional Mohr-Coulomb yield function

(63) $$f_{MC}=\mu \;p_{n}+\sqrt{\tau_{dt}^{2}+\tau_{dh}^{2}},$$

where μ is a friction coefficient and $p_{n}$ is the normal pressure on the interface. To take into the account the initially induced pressure from shrinkage of the concrete during the drying process after the production, the material parameter $p_{0}$ is introduced. To this end, the effective normal pressure can be phenomenologically assumed as

(64) $$p_{n}={\langle p_{0}+\sigma_{dn}\rangle }_{-}\;.$$

Furthermore, a plastic potential

(65) $$g=\sqrt{\tau_{dt}^{2}+\tau_{dh}^{2}}$$

is defined, describing, together with a plastic multiplier λ, the evolution of $s_{di}$ by the following flow rule

(66) $${\dot{s}}_{di}=\dot{\lambda }\frac{\partial g}{\partial t_{d}}=\dot{\lambda }\left[\begin{array}{c} 0\\ \frac{\partial g}{\partial \tau_{dt}}\\ \frac{\partial g}{\partial \tau_{dh}} \end{array}\right].$$

Moreover, this evolution equation is solved under consideration of the Kuhn–Tucker conditions

(67) $$\dot{\lambda }\geq 0,\;f_{MC}\leq 0,\;\dot{\lambda }\;f_{MC}=0\;.$$

It is obvious from Equation (66) that the inelastic separations evolve only in tangential directions, where friction occurs.

The applied damage formulation of the RIA considers the irreversible failure mechanisms under combined Mode I and Mode II loading by an appropriate degradation function

(68) $$D=\max_{\mathrm{history}}\left\{\max\left(0,\;\min\left(1,\;\frac{1}{\eta }\cdot \frac{\beta }{1+\beta }\right)\right)\right\},$$

where the driving force β represents the relation of the current state of separation with respect to the states of damage initiation in Mode I $s_{0I}$ and Mode II $s_{0II}$, reading

(69) $$\beta =\sqrt{\frac{{\langle s_{n}\rangle }_{+}^{2}}{s_{0I}^{2}}+\frac{s_{t}^{2}+s_{h}^{2}}{s_{0II}^{2}}}-1\;.$$

Furthermore, a ductility parameter η∈[0,1] is introduced, which describes the relation between the separation state of damage initiation and the critical states of full decohesion $s_{cI}$ and $s_{cII}$ in the particular mode. This relation is assumed to be equal for both loading modes and can be written as

(70) $$\eta =1-\frac{s_{0I}}{s_{cI}}=1-\frac{s_{0II}}{s_{cII}}\;.$$

**Figure 7:**
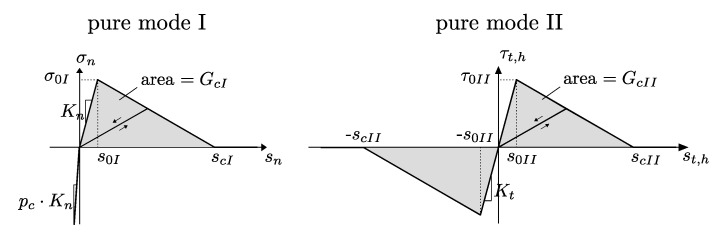
Bilinear interface damage evolution laws for mode I and mode II.

The resulting debonding behavior of the introduced TSL, under the absence of friction at pure Mode I and Mode II loading is schematically illustrated in Figure 7.

From this illustration, it is obvious that some of the introduced parameters of the damage formulation are correlated to each other and can also be expressed in terms of interfacial strength ($\sigma_{0I}$, $\tau_{0II}$) and fracture energies ($G_{cI}$, $G_{cII}$). In addition to the already described relations, the following dependencies and substitutions can be found according to [46,49]

(71) $$\begin{array}{cc} s_{cI}=\frac{2G_{cI}}{\sigma_{0I}}\; & ,\;s_{cII}=\frac{2G_{cII}}{\tau_{0II}}\;, \end{array}$$

(72) $$\begin{array}{cc} K_{n}=\frac{\sigma_{0I}}{s_{0I}}\; & ,\;K_{t}=\frac{\tau_{0II}}{s_{0II}}\;. \end{array}$$

Finally, the presented interface formulation can be easily implemented into a standard finite element software. Although, an eight node cohesive zone element with quadrilateral faces is considered, these elements can be adjusted for the application with triangular facets in a straightforward manner.

### 2.3. Calibration of Model Parameters

With defined material and interface formulations for the individual constituents of the composite, the introduced model parameters must be calibrated in order to sufficiently represent the properties of the particular materials. Here, the parameters of the corresponding models are identified by the related datasheets and the literature as well as experimental material tests, as it will be described in the following.

#### 2.3.1. Identification of Yarn Material Parameters

As explained in Section 2.2.1, the effective elastic properties can be calculated from the elastic parameters of the particular filaments and matrix material (coating). Here, the transversal isotropic properties of the HT carbon filaments are taken from [40]. The shear modulus $G_{m}$ of the applied Lefasol VLT-1 coating (produced by Lefatex-Chemie GmbH, Brüggen-Bracht, Germany), is measured in a torsional dynamic mechanical analysis by the Institute of Textile Machinery and High Performance Material Technology at TU Dresden. Since this coating is an SBR based polymer, the Poisson’s ratio is assumed to be close to 0.5. The used filament and matrix parameters are summarized in Table 1.

From the datasheet in [36], the effective Young’s modulus of the warp and weft yarn in longitudinal direction is given as $E_{||\mathrm{warp}}=170$ GPa and $E_{||\mathrm{weft}}=152$ GPa. Consequently, the corresponding fiber volume fractions of the warp and weft yarn can be calculated according to Equation (2), which yields $\varphi_{\mathrm{warp}}=0.737$ and $\varphi_{\mathrm{weft}}=0.658$. Finally, the effective stiffness matrix of the warp and weft yarns is computed, as it is described in Section 2.2.1. The effective stiffness matrices $C_{\mathrm{warp}}$ and $C_{\mathrm{weft}}$ are rotated, so that their preferential direction is oriented in global *x*- and *y*-directions, as shown in Figure 4.

#### 2.3.2. Identification of Concrete Material Parameters

For the investigated TRC in this study, a fine grained concrete matrix named TUDALIT-TF10- PAGEL, according to [36], is considered. Due to the complexity of the applied concrete material model, described in Section 2.2.2, some parameters are chosen according to similar concrete mixtures described in the literature and others are identified by experimental tests. However, in [50], the relation $c=l^{2}$ is derived, where *l* is the characteristic length scale parameter of the material. Following [51,52], the characteristic length scale for concrete is proportional to the maximum aggregate size. For the concrete at hand, the maximum aggregate size is 1 mm and, thus, $c=l^{2}=1$ mm${}^{2}$ is assumed. However, the eccentricity parameter is determined according to [53] as e=0.51. The other material parameters are identified by experimental tests, as it will be described in the following. Furthermore, the following relations between the uniaxial compressive strength $f_{uc}$ and some of the model parameters are used, based on [43]

(73) $$\begin{array}{c} f_{bc}=1.15f_{uc}, \end{array}$$

(74) $$\begin{array}{c} f_{ut}=1.4{\left(f_{uc}/10\right)}^{2/3}, \end{array}$$

(75) $$\begin{array}{c} \alpha =\frac{\sqrt{3}\left(f_{bc}-f_{uc}\right)}{2f_{bc}-f_{uc}}, \end{array}$$

(76) $$\begin{array}{c} \sigma_{0}=\left(1/\sqrt{3}-\alpha /3\right)f_{uc}, \end{array}$$

(77) $$\begin{array}{c} \sigma_{V}^{T}=-f_{uc}/3, \end{array}$$

(78) $$\begin{array}{c} T_{0}=f_{ut}/4. \end{array}$$

**Figure 8:**
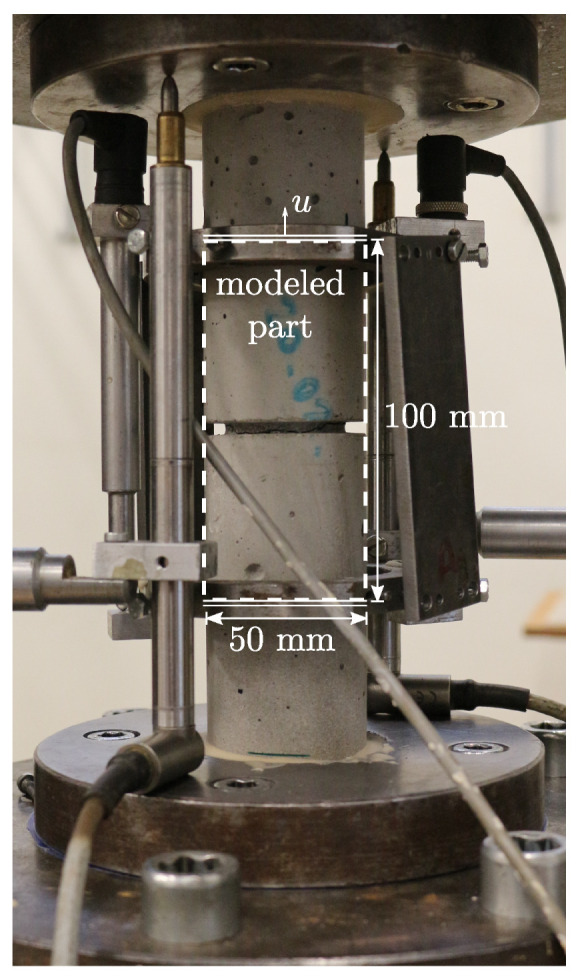
Experimental setup of tension test with notched cylindrical specimen.

**Figure 9:**
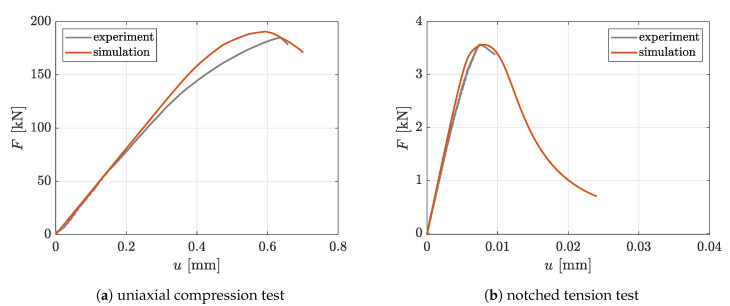
Reaction force vs. displacement curves of calibration experiments.

The conducted experimental investigations include uniaxial compression tests on unnotched cylindrical specimens and uniaxial tension tests on notched cylindrical specimens. Both specimen types have a diameter of 50 mm. The height of the compression specimen is 170 mm. Since the compression specimen is tested with polished steel plates of the machine, friction is assumed to be negligibly small and a uniform uniaxial test configuration is considered. The experimental setup of the notched tension test is shown in Figure 8. The notch has a height of 3 mm and a depth of 5 mm. Please note that the boundary effects of the glued connection between the specimen and the testing machine are assumed to be negligible. With this assumption, only the part between the gauge length of the inner inductive displacement sensors, with a height of 100 mm, can be taken into account by prescribing the measured displacements as boundary conditions in a vertical direction, as illustrated in Figure 8. Due to the symmetrical setup in this case, only an eighth with corresponding symmetry conditions is taken into consideration. The finally calibrated material parameters are summarized in Table 2. With this set of parameters, good agreement with respect to experimental results is achieved, as can be seen from the corresponding force displacement characteristics depicted in Figure 9.

#### 2.3.3. Identification of Interface Parameters

**Figure 10:**
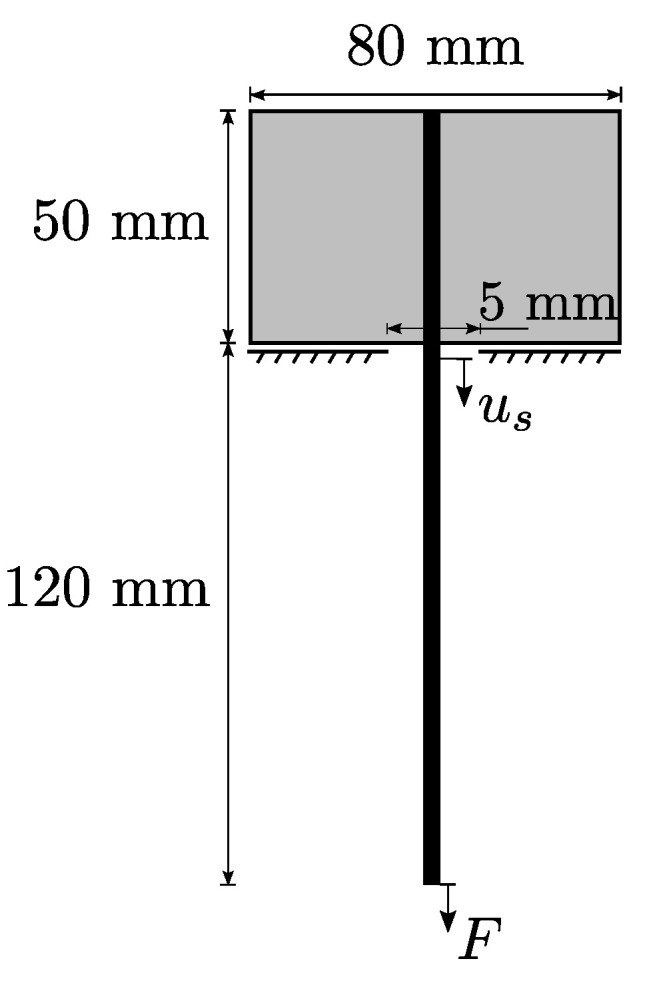
Schematical setup of simulated yarn pullout experiment.

**Figure 11:**
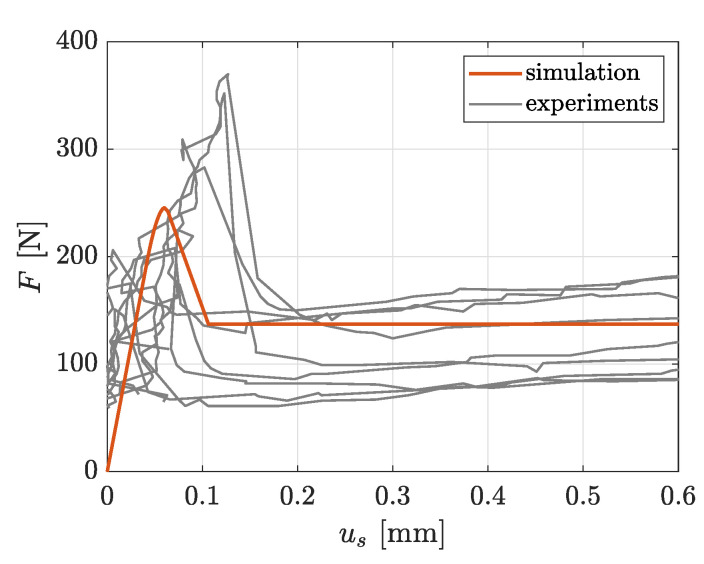
Force vs. slip curve of the experimental and modeled warp yarn pullout test.

For the calibration of the cohesive zone material parameters introduced in Section 2.2.3, experimental yarn pullout tests of TUDALIT-BZT2-V.FRAAS warp yarns embedded in TUDALIT-TF10-PAGEL concrete are conducted. The experimental setup is adopted from [54]. These tests are designed in such a way that a single yarn is embedded in two concrete blocks of different height, where the yarn is pulled out from the smaller block, due to the shorter embedment length. It is noteworthy to mention that the concrete and the warp yarn are modeled as linear elastic with the previously identified elastic parameters, since no damage of the concrete blocks or the yarn is observed in the experiments. It is also observed that the pullout only takes place in the smaller block. Therefore, just the smaller concrete block and the free yarn length are considered in the simulation. The schematic setup of the simulated yarn pullout experiment is depicted in Figure 10. During the experiment, the pullout force *F* and the displacement $u_{s}$ (called slip) of the yarn in a distance of 1 mm from the concrete surface is measured. Due to symmetries within the setup, just a quarter of the geometry is modeled in combination with according symmetry conditions. With these assumptions, a subsequent FE model, including cohesive zone elements at the material interface, is derived from the experimental setup. The finally calibrated parameters of the interface formulation are summarized in Table 3. The achieved simulation results show the expected phenomena such as debonding, decohesion as well as friction and are in good agreement with the experimental measurements, as shown in Figure 11. Since the weft yarn consists of the same filaments and coating, it is assumed that the interfacial behavior between concrete and weft yarn is equal to the interfacial behavior of the warp yarns.

## 3. Numerical Material Testing

At this point, the constructed TRC-RVE can be used in order to characterize the mechanical behavior of TRC by numerical material tests (NMT). With a proper constructed RVE defined, NMT offer several advantages compared to experimental investigations. Beside the lower costs, NMT enable a detailed insight into the micro- or mesomechanical behavior at clearly defined loading states as well as the derivation of the effective macroscopic behavior.

Here, a strain driven testing approach is used, where representative loading cases are imposed on the RVE by according strain states. Note that, in the case of TRC, the separation of scales is only fulfilled in the plane of the reinforcement layer (textile plane), cf. Figure 1. Therefore, only in-plane loading cases are considered in this work, namely tension in warp and weft direction as well as in-plane shear loading. As a consequence, the top and bottom of the TRC-RVE are treated as free boundaries with homogeneous Neumann boundary conditions. Following [55,56,57], symmetrical RVE can be further reduced by making use of symmetry and periodicity conditions. Hence, due to the two symmetry planes in the TRC-RVE, as shown in Figure 3, the NMT of normal loading conditions (e.g., uniaxial or biaxial loading), can be reduced to a quarter of the constructed RVE. Furthermore, in this contribution, a strain-based homogenization scheme is used, i.e., a predefined macroscopic strain state $\bar{\epsilon }=\left[{\bar{\epsilon }}_{xx},{\bar{\epsilon }}_{yy},{\bar{\gamma }}_{xy}\right]$ is imposed to the RVE via PBC (and the use of symmetries) and the homogenized macroscopic stress state $\bar{\sigma }=\left[{\bar{\sigma }}_{xx},{\bar{\sigma }}_{yy},{\bar{\sigma }}_{xy}\right]$ is observed, as described in [22,23,24,25]. The homogenized stress is calculated by the volume average of the mesoscopic stresses in the RVE $\Omega_{RVE}$ and reads

(79) $$\bar{\sigma }=\frac{1}{V_{RVE}}\int_{\Omega_{RVE}}\sigma \;d\Omega_{RVE},$$

where $V_{RVE}$ is the volume of the RVE. In addition, the distribution of the effective mesoscopic concrete damage (see Equation (33)) is analyzed, which is given as

(80) $$d^{meso}=\frac{1}{4\pi }\int_{\Omega }d^{mic}\;d\Omega \;.$$

### 3.1. Tensile Loading in Warp and Weft Direction

**Figure 12:**
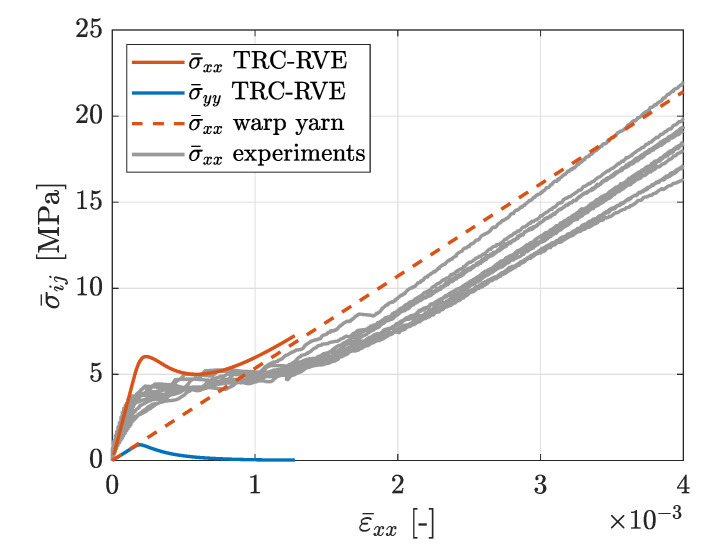
Effective macroscopic stress–strain relation due to tensile loading in warp yarn direction with prescribed strain $\bar{\epsilon }=\left[{\bar{\epsilon }}_{xx},{\bar{\epsilon }}_{yy}=0,{\bar{\gamma }}_{xy}=0\right]$.

The reinforcements in concrete are designed to compensate for the relatively low tensile strength and ductility of concrete. In particular, brittle cracking of the concrete matrix activates the reinforcement structure and the subsequent crack-bridging effects lead to an improved load bearing behavior of the composite material. Therefore, the mechanical behavior of textile reinforced concrete at tensile loading is of great interest with special regard to the evolving degradation characteristics and load bearing mechanisms. For that purpose, the tensile behavior of the constructed TRC-RVE in the direction of the different yarns is investigated. First, the RVE behavior in warp yarn direction (global *x*-direction) at uniaxial tensile loading $\bar{\epsilon }=\left[{\bar{\epsilon }}_{xx},{\bar{\epsilon }}_{yy}=0,{\bar{\gamma }}_{xy}=0\right]$ is analyzed. The evolved mesoscopic damage distribution of this particular loading case is shown in Figure 12. It is obvious that one major damage band evolves perpendicular to the main loading direction and along the weft yarns. Thus, due to the local stress concentrations induced by the weft yarns and the yarn crossing, the location and orientation of the damage band evolves as expected. The corresponding homogenized effective stresses are shown in Figure 12. Since a uniaxial strain state is imposed on the TRC-RVE, the transverse strain-blocking effect leads to a biaxial stress state. Consequently, in addition to the stress components in the *x*-direction, stresses in the *y*-direction are induced. From the shown macroscopic stress–strain behavior, it can be seen that the RVE behaves initially linear elastic with an effective composite stiffness. With increasing load, local damage zones evolve and coalesce, leading to a local and later to a macroscopic softening behavior with peak stresses of ${\bar{\sigma }}_{xx}=6.03$ MPa and ${\bar{\sigma }}_{yy}=0.91$ MPa. The further evolution of damage and the subsequent exponential degradation of the concrete matrix within the damage band leads to the activation of the warp yarns. Once the damage band has fully evolved across the whole RVE, the concrete matrix looses its load bearing capacity in the direction of loading and all loads are carried by the activated yarns. Consequently, the macroscopic load bearing capacity of the composite at this state is governed by the linear elastic behavior of the warp yarns. Therefore, it is obvious that the analytical macroscopic linear elastic response of the yarns, shown in Figure 12, is the limit state of this loading case, to which the effective response of the fully damaged RVE must converge. This analytical limit case in warp direction is defined as

(81) $${\bar{\sigma }}_{xx,\mathrm{warp}}=E_{||\mathrm{warp}}\;\psi_{\mathrm{warp}}\cdot {\bar{\epsilon }}_{xx},$$

where $\psi_{\mathrm{warp}}$ is the warp yarn volume fraction, as given in Section 2.1. However, the characteristic transition process in the load bearing behavior of TRC is clearly captured in the effective stress strain relation of the TRC-RVE, indicated by post peak softening followed by asymptotic stress increase towards the purely linear elastic yarn behavior in loading direction. Moreover, the decreasing load bearing of the concrete matrix is due to the reduction of the transverse strain-blocking effect, which leads to the asymptotic decrease of ${\bar{\sigma }}_{yy}$.

**Figure 13:**
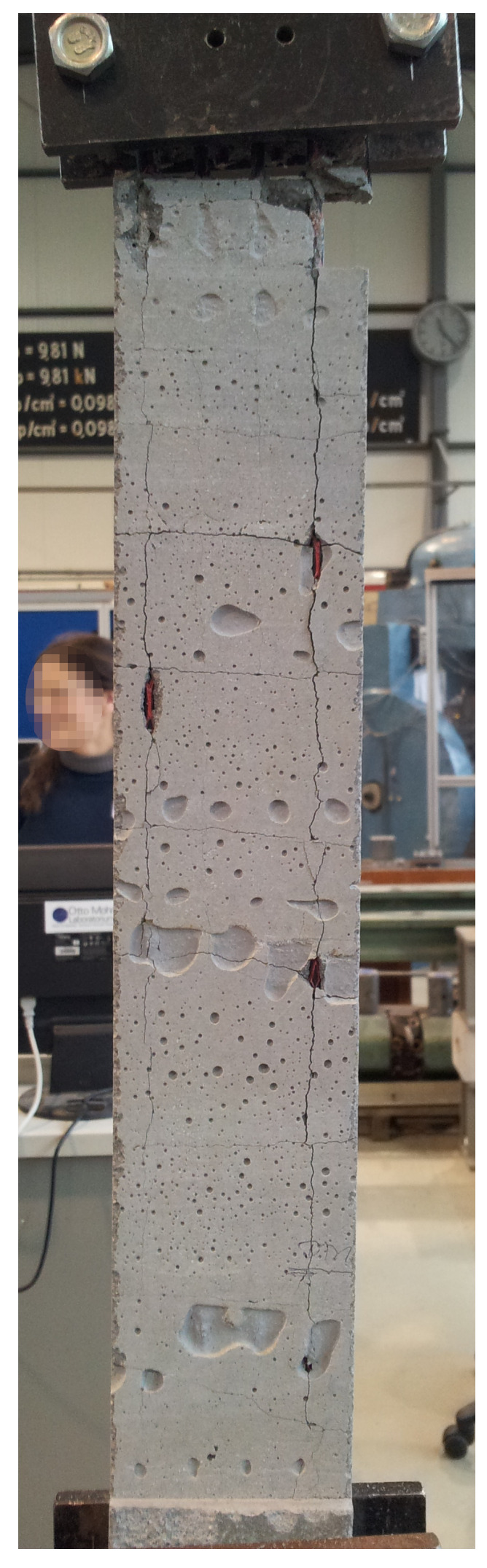
Experimental setup of TRC tensile test specimens according to [36], (photo by H. Michler (Institute of Concrete Structures, TU Dresden)).

In order to verify the constructed TRC-RVE, in Figure 12, the achieved numerical results in warp direction are compared to experimental results of tensile test specimens according to [36] and reported in [37]. The experimental setup is shown in Figure 13.

It should be clearly pointed out that the NMT procedure of the constructed TRC-RVE is not designed to model this particular tensile experiment, since some of the considered assumptions within the NMT framework are not fulfilled in the experiment. Firstly, the NMT framework is a strain based procedure, while the experiment is designed to apply a uniaxial stress state to the specimen. Secondly, the fundamental concept of scale separation does not hold for the experimental specimen, since it contains just four longitudinal warp yarns and is strongly influenced by boundary effects, especially at the clamping (see Figure 13). These boundary effects are not captured within the introduced homogenization scheme. Finally, the idealizations within the construction of the RVE do not consider any geometrical or material imperfections, e.g., voids, and lead, in combination with the assumed periodicity, to a uniform behavior in every unit cell of the virtual composite. Hence, the detailed modeling of this particular experimental setup is not the aim of this study. Nevertheless, the main characteristics of the constitutive composite behavior remain comparable and give a valuable assessment of the primary mechanical properties.

Considering these remarks, the effective macroscopic behavior of the TRC-RVE is in good agreement with the observed mechanical characteristics of the shown experimental setup, as it can be seen in Figure 12. In particular, the initial composite stiffness as well as the linear elastic limit state of the yarns match quite well. A slightly different behavior is observed at the beginning of the damage initiation of the concrete matrix. Here, the RVE shows a peak in combination with softening in the macroscopic stress strain relation, while the experiments show a monotonically increasing transition at a lower stress level. This difference could be caused by the weakening of the concrete matrix due to boundary effects and imperfections, since these effects are not captured by the idealized RVE. However, the transition to yarn dominated load bearing behavior is very close to the experimental observations. Please note that the used geometrical as well as material parameters are identical to the calibrated parameters in the previous sections. Therefore, the numerical results of the final RVE are not achieved by a curve fitting procedure. In conclusion, it can be stated that the constructed TRC-RVE captures the major mechanical characteristics of the particular composite and is, hence, considered as representative for further investigations.

**Figure 14:**
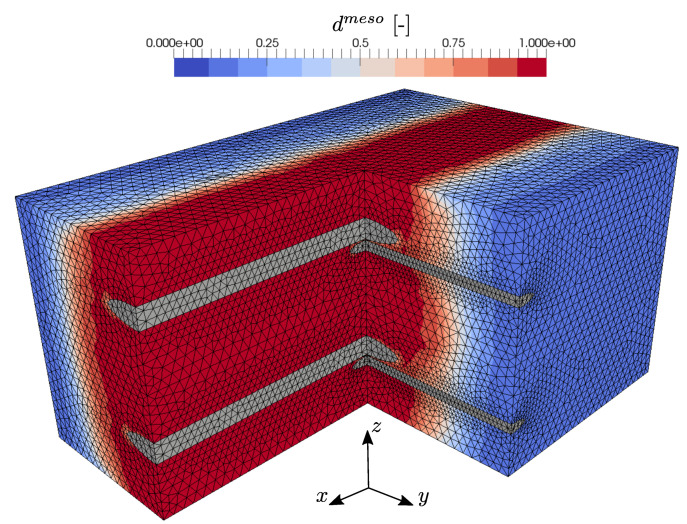
Evolved effective mesoscopic damage distribution due to tensile loading in warp yarn direction with prescribed strain $\bar{\epsilon }=\left[{\bar{\epsilon }}_{xx},{\bar{\epsilon }}_{yy}=0,{\bar{\gamma }}_{xy}=0\right]$.

**Figure 15:**
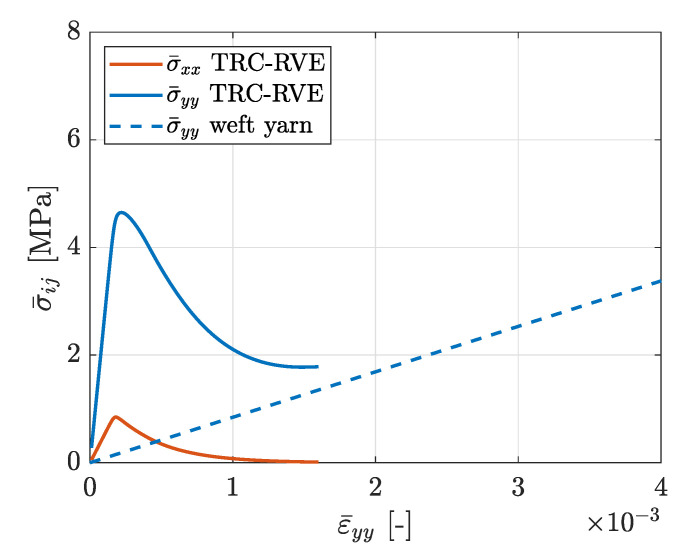
Effective macroscopic stress–strain relation due to tensile loading in weft yarn direction with prescribed strain $\bar{\epsilon }=\left[{\bar{\epsilon }}_{xx},{\bar{\epsilon }}_{yy}=0,{\bar{\gamma }}_{xy}=0\right]$.

Next, the mesomechanical behavior of the TRC-RVE at tensile loading in weft direction is analyzed by imposing the uniaxial strain state $\bar{\epsilon }=\left[{\bar{\epsilon }}_{xx}=0,{\bar{\epsilon }}_{yy},{\bar{\gamma }}_{xy}=0\right]$ to the RVE. The evolved mesoscopic damage distribution, depicted in Figure 14, as well as the effective macroscopic constitutive relation, shown in Figure 15, reveal an analogous behavior to the tensile characteristics in warp direction. As expected from the previous loading case, a damage band evolves perpendicular to the main loading direction and along the warp yarns, due to the strong stress concentrations induced by the warp yarn and the yarn crossings. As it can be seen from Figure 15, the transverse strain-blocking effect induces ${\bar{\epsilon }}_{xx}$ stresses, transversal to the main loading direction. Moreover, the RVE behaves initially linear elastic until local damage zones evolve and coalesce at the yarn crossings, leading to the macroscopic peak stresses and a post peak softening behavior, analog to tensile loading in the warp direction. In this case, the resulting peak stresses yield ${\bar{\sigma }}_{xx}=4.95$ MPa and ${\bar{\sigma }}_{yy}=0.85$ MPa and are smaller compared to the peak stresses in warp direction, which is mainly caused by more pronounced weakening of the load carrying cross-section and the lower stiffness of the weft yarns. Moreover, the lower stiffness and the smaller cross-section of the weft yarns also lead to a more dominant softening behavior of the TRC and a considerably slower transition to the elastic limit, given as

(82) $${\bar{\sigma }}_{yy,\mathrm{weft}}=E_{||\mathrm{weft}}\;\psi_{\mathrm{weft}}\cdot {\bar{\epsilon }}_{yy}.$$

In addition, in this case, the decreasing load bearing of the concrete matrix is the reduction of the transverse strain-blocking effect, which leads to the asymptotic decrease of ${\bar{\sigma }}_{xx}$.

### 3.2. Biaxial Tensile Loading

**Figure 16:**
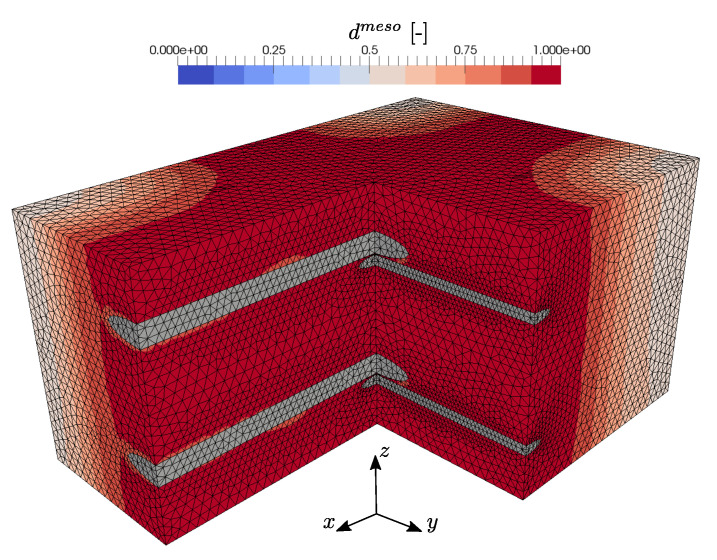
Evolved effective mesoscopic damage distribution due to biaxial tensile loading with prescribed strain $\bar{\epsilon }=\left[{\bar{\epsilon }}_{xx}={\bar{\epsilon }}_{bt},{\bar{\epsilon }}_{yy}={\bar{\epsilon }}_{bt},{\bar{\gamma }}_{xy}=0\right]$.

**Figure 17:**
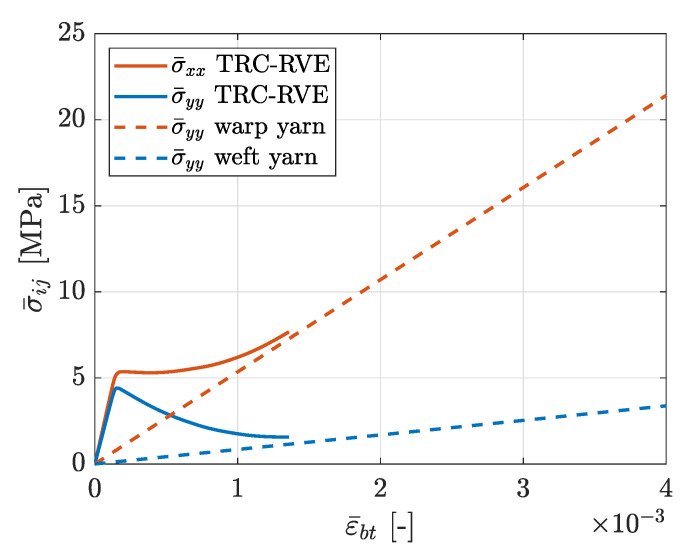
Effective macroscopic stress–strain relation due to biaxial tensile loading with prescribed strain $\bar{\epsilon }=\left[{\bar{\epsilon }}_{xx}={\bar{\epsilon }}_{bt},{\bar{\epsilon }}_{yy}={\bar{\epsilon }}_{bt},{\bar{\gamma }}_{xy}=0\right]$.

After the numerical testing of uniaxial loading scenarios, the mesomechanical RVE behavior at equibiaxial tensile loading is analyzed by an imposed macroscopic strain state $\bar{\epsilon }\;=\;\left[{\bar{\epsilon }}_{xx}\;=\;{\bar{\epsilon }}_{bt},{\bar{\epsilon }}_{yy}\;=\;{\bar{\epsilon }}_{bt},{\bar{\gamma }}_{xy}\right]$. The distribution of effective mesoscopic damage in the concrete matrix for this case is depicted in Figure 16. As expected from the previous loading cases, two prominent crossing damage bands evolve along the warp and weft yarns. While in the uniaxial states, undamaged parts of the concrete matrix remain, in this case, effective damage evolves in the entire concrete matrix. The corresponding macroscopic constitutive stress–strain relation is shown in Figure 17. It can be seen that, after the initial linear elastic RVE response, the previously observed transition in the load bearing behavior occurs, due to the degradation of the concrete. It is interesting that, compared to the uniaxial loading in warp direction, no pronounced peak occurs in the resulting normal stress ${\bar{\sigma }}_{xx}$, which is caused by the biaxial loading state and the subsequent damage initiation at a lower stress level of ${\bar{\sigma }}_{xx}=5.36$ MPa. In contrast to that, the resulting stress ${\bar{\sigma }}_{yy}$ shows the expected post-peak softening behavior with a peak stress of ${\bar{\sigma }}_{yy}=4.41$ MPa. Finally, both stress components tend asymptotically towards the corresponding elastic limit state of the warp and weft yarns, respectively.

### 3.3. In-Plane Shear Loading

**Figure 18:**
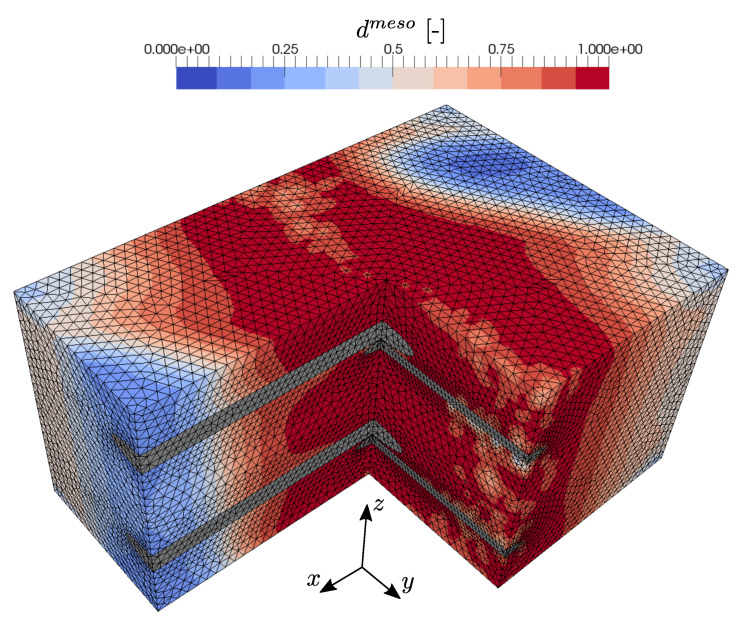
Evolved effective mesoscopic damage distribution due to in-plane shear loading with prescribed strain $\bar{\epsilon }=\left[{\bar{\epsilon }}_{xx}=0,{\bar{\epsilon }}_{yy}=0,{\bar{\gamma }}_{xy}\right]$.

**Figure 19:**
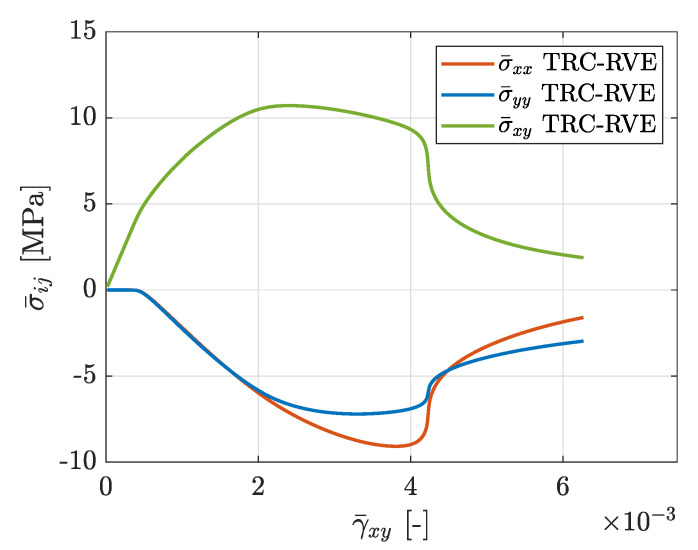
Effective macroscopic stress–strain relation due to in-plane shear loading with prescribed strain $\bar{\epsilon }=\left[{\bar{\epsilon }}_{xx}=0,{\bar{\epsilon }}_{yy}=0,{\bar{\gamma }}_{xy}\right]$.

Another important aspect for the design of concrete structures reinforced by TRC is the mechanical composite behavior at in-plane shear loading, since it leads to a strong interaction of different local phenomena and induces complex damage mechanisms. For the characterization of this intricate loading case, the TRC-RVE is loaded in a pure shear mode imposed by the corresponding macroscopic strain state $\bar{\epsilon }=\left[{\bar{\epsilon }}_{xx}=0,{\bar{\epsilon }}_{yy}=0,{\bar{\gamma }}_{xy}\right]$. The distribution of effective mesoscopic damage in the concrete matrix for this case is depicted in Figure 18. It can be seen that a point symmetric damage distribution evolves, where first one primary inclined damage band evolves across the center of the RVE to opposite corners leading to ductile degradation. Furthermore, two secondary damage bands evolve in the other corners, which are induced by the periodic boundary conditions of the nonlocal DOF and, hence, capture the propagation of the primary damage bands from the neighboring RVE. The concrete in between these bands remains undamaged. In the further evolution of the previously inclined damage band, two dominant damage bands form along the weft yarns at ${\bar{\gamma }}_{xy}=4.2\cdot 10^{-3}$, leading to transition from ductile to brittle failure behavior. The corresponding effective constitutive stress–strain relation is shown in Figure 19. In comparison to the previous tensile loading cases, the macroscopic behavior at shear loading is more ductile with a pronounced hardening phase after the initiation of damage and a broader stress peak with a maximum value of ${\bar{\sigma }}_{xy}=10.68$ MPa. Furthermore, ductile softening behavior can be observed after the peak followed by steep brittle degradation and the subsequent exponential softening, caused by the formation of the two damage bands along the weft yarns out of the previously inclined damage band. It is also interesting that, with the beginning of the nonlinear RVE behavior, normal stresses ${\bar{\sigma }}_{xx}$ and ${\bar{\sigma }}_{yy}$ are induced, due to the evolution of plastic strains.

## 4. Conclusions and Outlook

The work at hand presents a comprehensive approach to the numerical mesoscale analysis of textile reinforced concrete by a representative volume element applied, but not limited to, TRC described in [36]. Firstly, the choice of an adequate RVE based on the idealization of the real TRC mesostructure is explained and the according geometrical dimensions of the RVE are derived. Furthermore, sufficient constitutive formulations are presented, accounting for the dominant characteristics of the textile yarns and the concrete as well as their interactions at the material interfaces. In particular, the textile yarns are modeled as linear elastic transversal isotropic unidirectional layers, based on the elastic properties of the filaments and the yarn coating. The shown material model for concrete accounts for the complex plasticity and damage behavior at multiaxial loading scenarios. The mesomechanical interactions of these constituents are modeled by an interface formulation, which takes into account debonding and frictional effects as well as contact. In a further step, the introduced constitutive descriptions are calibrated by corresponding datasheets and individual experimental results. By the finally constructed TRC-RVE, the mesoscopic damage evolution as well as the effective mechanical behavior at the macroscale are analyzed and discussed in several relevant loading cases by a numerical material testing procedure based on a first order homogenization approach. The numerical results of one representative loading case (tension in warp direction) are compared to experimental results. It is shown that the constructed TRC-RVE is able to capture the dominant mechanical characteristics of the composite under the considered assumptions of the analysis.

Finally, it can be concluded that the presented modeling approach and the mesomechanical analysis of TRC can give deeper understanding and valuable insight into the mesostructural interactions. Furthermore, it enables the derivation of the effective macroscopic constitutive behavior of the composite. In addition, the influence of geometrical as well as material design parameters can be easily captured in order to find an optimal mesostructural setup for a certain application. Moreover, by considering the uncertainties within the mesostructural setup, the presented approach can be used as a basis for robust design optimization of the composite properties, as demonstrated in [58]. Another very interesting aspect is the mesomechanical behavior at transient loading cases, e.g., impact scenarios, which could be addressed by enhancing the presented NMT framework by the approaches described in [59] or [60]. Research regarding the mentioned robust design optimization as well as investigation of the transient TRC characteristics is the scope of ongoing studies.

## Figures and Tables

**Table 1:** Elastic parameters of yarn constituents.

Carbon Filaments	Lefasol VLT-1 Coating
$E_{f\|}$ = 230,000 MPa	$E_{m}=G_{m}\left(2+2\nu_{m}\right)$ = 1802.9 MPa
$E_{f⊥}$ = 28,000 MPa	
$G_{f⊥\|}$ = 50,000 MPa	$G_{m}$ = 605 MPa
$\nu_{f⊥\|}$ = 0.23	$\nu_{m}$ = 0.49
$\nu_{f⊥⊥}$ = 0.259	

**Table 2:** Identified material parameters of concrete material model.

*E* [GPa]	ν [-]	$f_{\mathrm{uc}}$ [MPa]	$D_{h}$ [MPa${}^{2}$]	$R_{t}$ [-]	$\sigma_{V}^{C}$ [MPa]	*R* [-]	*W* [-]	$D_{c}$ [1/MPa]	*e* [-]	$A_{s}$ [-]	$\gamma_{c0}$ [-]	$\gamma_{t0}$ [-]	$\beta_{t}$ [-]	$\beta_{c}$ [-]	*c* [mm${}^{2}$]	*m* [-]
35	0.2	105	72,500	0.5	−80	1	0.1325	0.00125	0.51	1.9	$1.5\cdot 10^{-4}$	0	4000	3500	1	2

**Table 3:** Identified parameters of interface element formulation.

$\sigma_{0I}$ [MPa]	$G_{\mathrm{cI}}$ [N/mm]	$\tau_{0\mathrm{II}}$ [MPa]	$G_{\mathrm{cII}}$ [N/mm]	η [-]	μ [-]	$p_{0}$ [MPa]
0.9	0.05	0.8	0.3	0.45	0.6	0.7
